# Temperature Response of Planktonic Microbiota in Remote Alpine Lakes

**DOI:** 10.3389/fmicb.2019.01714

**Published:** 2019-07-31

**Authors:** Yiming Jiang, Haiying Huang, Tianli Ma, Jinlong Ru, Stephan Blank, Rainer Kurmayer, Li Deng

**Affiliations:** ^1^Institute of Virology, Helmholtz Zentrum München, German Research Center for Environmental Health, Neuherberg, Germany; ^2^Institute of Virology, Technical University of Munich, Munich, Germany; ^3^Research Department for Limnology, Mondsee, University of Innsbruck, Innsbruck, Austria

**Keywords:** high mountain lakes, climate change, growing seasons, nutrient level, metabarcoding, richness, activation energy

## Abstract

Alpine lakes are considered pristine freshwater ecosystems and sensitive to direct and indirect changes in water temperature as induced by climate change. The bacterial plankton constitutes a key component in the water column and bacterial metabolic activity has direct consequences for water quality. In order to understand bacterial response to global temperature rise in five alpine lakes located in the Austrian Alps (1700–2188 m a.S.L.) water temperature was compared within a decadal period. Depth-integrated samples were characterized in community composition by 16S rDNA deep-amplicon sequencing early [56 ± 16 (SD) days after ice break up] and later (88 ± 16 days) in the growing season. Within the 10 years period, temperature rise was observed through reduced ice cover duration and increased average water temperature. During the early growing season, the average water temperature recorded between circulation in spring until sampling date (WAS), and the day of autumn circulation, as well as chemical composition including dissolved organic carbon influenced bacterial community composition. In contrast, only nutrients (such as nitrate) were found influential later in the growing season. Metabolic theory of ecology (MTE) was applied to explain the dependence of taxonomic richness on WAS in mathematical terms. The calculated activation energy exceeded the frequently reported prediction emphasizing the role of WAS during early growing season. Accordingly, the relative abundance of predicted metabolism related genes increased with WAS. Thus, the dominant influence of temperature after ice break up could be explained by overall climate change effects, such as a more intense warming in spring and an overall higher amplitude of temperature variation.

## Introduction

Climate change, which is mainly caused by anthropogenic activities, is a global issue. Since 1850, the Earth’s surface temperature has increased by 0.76°C, and it is expected to increase by another 1.1–6.4°C by the end of this century (Stocker, 2014). Ongoing climate change has been shown to cause a rapid change in ecological communities, thereby threatening ecosystem functioning and services (Jassey et al., 2013). Mountain regions are suffering more from warming than the global average (Beniston et al., 1997; Sommaruga-Wögrath et al., 1997; Parker et al., 2008). Over the past century, the annual average temperature in the Alps has risen by about 2°C, which is on average twice as high as estimated for the northern hemisphere (Djukic et al., 2013; Weckström et al., 2016). The alpine lake ecosystem is affected by global climate change through thawing of ice, melting of frozen soils, altered snow packing, shortened ice cover duration, and increasing summer water temperatures, thereby affecting biodiversity and functioning of water cycles and the microclimate (Holzapfel and Vinebrooke, 2005; Parker et al., 2008). Traditionally mountain lake ecosystems are highly sensitive to global warming, functioning as sentinels of climate change (Kamenik et al., 2001; Adrian et al., 2009; Williamson et al., 2009). Although various studies have documented the responses of plant and animal biodiversity to temperature, less studies have examined microbial responses to climate warming (Zhou et al., 2016; Guo et al., 2018).

One current challenge is to determine how environmental parameters (in)directly related by climate change affect the structure and diversity of the microbiota in lakes. Various factors have been elucidated, for example, studies have shown that climate warming impacts on alpine’s lake bacterioplankton community structure through affecting the turbidity shift along with the glacier retreat (Peter and Sommaruga, 2016), or through changing the surrounding soil characteristics (Rofner et al., 2017). Besides, it is well established that temperature is a key driver in microbiota biodiversity construction, as temperature directly regulates metabolic rates and biochemical processes (Gillooly et al., 2001; Brown et al., 2004; Zhou et al., 2016). The metabolic theory of ecology (MTE) addresses the relationships between ecosystem properties, body size, and metabolism (Brown et al., 2004). The dependence of species richness on temperature is based on the energetic equivalence rule (Allen et al., 2002), assuming that the total energy flux of a population per unit area does not depend on body size. It is predicted that at higher temperature, each species has fewer individuals (because of faster growth) but the total number of individuals is the same. In other words, with increasing temperature the richness is increasing, since more species are present. Although the MTE was established for macroorganisms in the last decade, for the microbiota in soil this relationship has also been found (Zhou et al., 2016).

In this study we compared average water temperatures and ice cover duration observed during two summers in lakes located in the alpine zone of the Austrian Alps to records from 10 years earlier. We expected that based on the general understanding of climate change water temperature should show an increase in relation to corresponding air temperature while ice cover duration should decline. In a second step, we aimed to understand how the microbiota in alpine lakes respond to the temperature changes and other environmental factors occurring seasonally during two consecutive years. We hypothesized that besides water temperature regional factors influenced by the geology in the catchment (e.g., Kamenik et al., 2001) should be of major influence. In a third step MTE was applied to explain the dependence of taxonomic richness on the bacterial metabolic activity. We expected the so-called activation energy E_a_ (calculated from the slope between temperature and taxa richness) in a range of the previously observed and more frequently reported -0.65 prediction equivalent to a Q_10_ of ∼2.5 (Stegen et al., 2009; Zhou et al., 2016). Taking into account the overall climate change scenario on temperature rise in the Alps we speculate on the role of climate change effects.

## Materials and Methods

### Study Sites and Topographic Parameters

**FIGURE 1:**
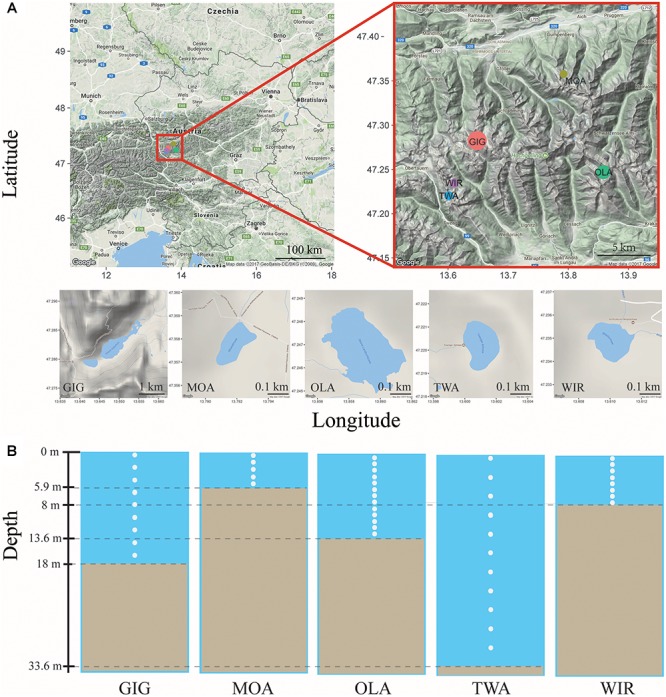
**(A)** Map of study area and the five alpine lakes in the “Niedere Tauern” region of the Austrian Alps. **(B)** Depth-integrated sampling of water column. The subsamples were taken at every 1 m (MOA, OLA, and WIR), every 2 m (GIG), or every 3 m (TWA) from surface. The maps were generated with “ggmap” in R, a package based on Google Maps.

Five alpine lakes in the “Niedere Tauern” region of the eastern Alps, Austria (Weckström et al., 2016) [Unterer Giglachsee (GIG), Moaralmsee (MOA), Oberer Landschitzsee (OLA), Twenger Almsee (TWA), and Wirpitschsee (WIR)], were selected as sampling sites (Figure 1A). The lakes were located along an altitude gradient ranging between 1700 and 2120 m above sea level (a.S.L.) in the alpine zone either above the treeline (WIR) or above the timberline (others). MOA was the smallest lake with an area of 2.1 ha and max depth of 5.9 m; the lake area and max depth of other lakes were 3.1–16.8 ha and 8–33.6 m, respectively (Table 1). Maps of the five lakes were generated with R using package “ggmap”. In addition, meteorological data recorded from four relevant stations were used to compare changes in temperature and to analyze the potential influence of precipitation on the observed microbiota community composition: Sonnblick (3109 m a SL), Rudolfshütte (2317 m), Schmittenhöhe (1956 m), Obertauern (1772 m).

**Table 1:** Environmental characteristics (mean ± SD) for five alpine lakes.

Lake		Unterer Giglachsee GIG	Moaralmsee MOA	Oberer Landschitzsee OLA	Twenger Almsee TWA	Wirpitschsee WIR
Altitude	(m a.s.l)	1922	1825	2067	2118	1700
Lake area	(ha)	16.8	2.13	8.8	3.11	2.72
Max depth	(m)	18	6	14	33	8
YAWT (2010–2011)		5.43	4.90	6.06	5.34	6.41
YAWT (1998–1999)		5.25	4.46	5.05	4.61	5.34
ICD (2010–2011)		221.5	205	222	232.5	193
ICD (1998–1999)		221	223	224	240	209

Conductivity		74.15 ± 0.52^b^	28.83 ± 2.1^c^	14.15 ± 0.67^d^	72.4 ± 1.36^b^	85.25 ± 2.16^a^
pH		7.5 ± 0.42^a^	6.95 ± 0.58^ab^	6.19 ± 0.39^b^	7.36 ± 0.09^a^	7.46 ± 0.61^a^
Alkalinity	(μeq/L)	607.5 ± 12.29^b^	186.75 ± 11.18^d^	70.5 ± 3.87^e^	552.75 ± 5.74^c^	724.75 ± 27.44^a^
${\mathrm{NO}}_{3}^{–}$	(μM)	0.23 ± 0.09^d^	3.13 ± 0.17^a^	1.04 ± 0.04^c^	0.03 ± 0.03^d^	2.52 ± 0.37^b^
${\mathrm{SO}}_{4}^{2–}$	(μM)	60.63 ± 0.73^b^	29.06 ± 2.08^d^	21.67 ± 0.73^e^	76.35 ± 2.92^a^	52.71 ± 0.21^c^
Cl^-^	(μM)	5.07 ± 0.28^a^	3.94 ± 1.13^ab^	3.1 ± 0.85^b^	4.23 ± 0.56^ab^	5.63 ± 1.13
${\mathrm{NH}}_{4}^{+}$	(μM)	0.24 ± 0.1	0.22 ± 0.2	0.17 ± 0.1	0.18 ± 0.07	0.26 ± 0.05
Na^+^	(μM)	19.13 ± 0.43^b^	21.3 ± 0.87^b^	13.04 ± 1.3^c^	19.13 ± 0.43^b^	25.22 ± 1.74^a^
K^+^	(μM)	3.59 ± 0.26^c^	7.44 ± 0.51^a^	7.18 ± 0.26^a^	4.62 ± 0.26^b^	5.13 ± 0.77^b^
Mg^2+^	(μM)	90.91 ± 2.06^b^	11.52 ± 0.41^d^	5.76 ± 0^e^	121.76 ± 2.47^a^	69.11 ± 2.06
Ca^2+^	(μM)	271.75 ± 4^b^	112.75 ± 5.5^d^	49.5 ± 3.75^e^	224.75 ± 3.25^c^	347.75 ± 12.75^a^
TP	(μM)	0.14 ± 0.04^a^	0.1 ± 0.02^a^	0.08 ± 0.01^b^	0.11 ± 0.03^a^	0.08 ± 0.03
DOC	(μg/L)	858 ± 71.82^a^	444.75 ± 64.55^bc^	631 ± 44.79^bc^	590.25 ± 40.14^bc^	407 ± 179.25^c^
DN	(μg/L)	73.5 ± 16.86	209 ± 9.83	109.25 ± 14.38	218.5 ± 194.01	244.5 ± 127.25
DRSi	(μM)	0.08 ± 0.03	0.08 ± 0.03	10.84 ± 0.48	11.43 ± 2.22	17.84 ± 1.17

Chl a	(μg/L)	1.98 ± 0.34^a^	0.44 ± 0.72^b^	0.43 ± 0.26^b^	0.85 ± 0.61^ab^	0.35 ± 0.05^b^
Bacteria	(Counts/ml)	2.28 × 10^6^ ± 5.17 × 10^5a^	7.39 × 10^5^ ± 2.42 × 10^5b^	7.47 × 10^5^ ± 3.05 × 10^5b^	1.89 × 10^6^ ± 5.42 × 10^5ab^	9.14 × 10^5^ ± 3.44 × 10^5b^
Cyanobacteria	(Counts/ml)	1.05 × 10^5^ ± 8.93 × 10^4a^	9.63 × 10^2^ ± 2.79 × 10^2b^	4.71 × 10^2^ ± 1.62 × 10^2b^	1.17 × 10^4^ ± 7.81 × 10^3b^	2.93 × 10^3^ ± 2.61 × 10^3b^

Sampling Date^∗^	2010	W3Jul/W3Aug	W3Jul/W3Aug	W3Jul/W4Aug	W2Jul/W4Aug	W2Jul/W4Aug
	2011	W5Jul/W3Aug	W1Jul/W3Aug	W2Jul/W3Aug	W5Jul/W4Aug	W1Jul/W1Aug


*(i) Superscripts indicate significant difference among lakes (*p* < 0.05). (ii) ^∗^W… means the week of sampling in July or August.*

### Sampling

Depth-integrated water samples were taken in the years 2010 and 2011 during (i) early stage of the growing season, i.e., in July 26–56 ± 17–82 (min–mean ± SD–max) days after ice break up, and (ii) later stage of the growing season, i.e., in August 69–88 ± 16–116 days after ice break up. Depth-integrated samples were taken based on the maximum depth of each lake, i.e., were integrated by subsampling every meter in MOA, OLA, and WIR, every two meters in GIG, and every three meters in TWA (Figure 1B and Table 1). To minimize changes occurring during transport, samples (1.5–2 L) were prefiltered immediately on the shore using a hand vacuum pump through previously autoclaved glass fiber filters (GF/C) to remove eukaryotic algae and particle-associated bacteria. An aliquot of 100 mL of each sample was fixed using formaldehyde (2% v/v final concentration) and used for bacterial cell counting by DAPI staining according to standard conditions (Porter and Feig, 1980). One to three milliliters were filtered onto polycarbonate membrane filters (0.2 μm, Nuclepore Track-Etch membrane, Whatman, Dassel, Germany) and microbes were counted using epifluorescence microscopy at 1000-fold magnification (Axioplan, Zeiss, Oberkochen, Germany). Cyanobacteria were differentiated using autofluorescence. The glass fiber prefiltered water samples were filtered onto previously autoclaved nitrocellulose membranes (NC, pore diameter: 0.2 μm, Whatman, 1–2.1 L) using low-vacuum filtration (<0.4 bar), and the filters were transferred into Eppendorf tubes, immediately frozen in dry ice, and stored at -20°C until DNA extraction.

### Environmental Parameters

Water temperature was recorded at 2.5 m-depth in 2-h intervals between 26 September 1998 and 26 September 1999 (Schmidt et al., 2004) and in 4-h intervals between 1 September 2009 and 28 August 2012 using thermistors (MINILOG, Vemco Ltd., Bedford, NS, Canada). For each year and each lake, the calendar dates of spring mixing and autumn mixing were estimated by visual examination of individual temperature curves as described previously (Schmidt et al., 2004; Kamenik and Schmidt, 2005). The calendar day of circulation in autumn (CiA) was calculated from the date when the water temperature decreased to 4°C, while the calendar day of circulation in spring (CiS) was calculated from the first date when water temperature exceeded 4°C. The duration of ice cover (ICD) was calculated from the number of days in between CiA and CiS (Schmidt et al., 2004). The annual average water temperature (YAWT) over the entire year (including both the ice-free and ice-covered periods) and the average water temperature during the period between the calendar day of circulation in spring until the sampling date (WAS) were calculated.

Conductivity (Cond.) and pH were determined in the field using a multiprobe (YSI6920, YSI Environmental, Yellow Springs, OH, United States). The depth-integrated samples for chemical analyses were kept cool and analyzed within 2 days and included the following: Sulfate (${\mathrm{SO}}_{4}^{2–}$), chloride (Cl^-^), ammonium (${\mathrm{NH}}_{4}^{+}$), sodium (Na^-^), potassium (K^+^), magnesium (Mg^2+^), calcium (Ca^2+^), total phosphorus (TP), dissolved organic carbon (DOC), nitrate (${\mathrm{NO}}_{3}^{–}$), dissolved nitrogen (DN), and dissolved reactive silica (DRSi), were determined as described previously (Kamenik et al., 2001; Schmidt et al., 2004). The concentration of chlorophyll a (Chl a) was determined from depth-integrated sample aliquots filtered on GF/C filters and stored frozen until extraction using hot ethanol (International Organisation of Standardization [ISO], 1992).

### DNA Extraction

DNA was extracted and purified from bacteria collected on NC filters (as described above) using a NucleoSpin^®^ Soil DNA purification kit (MACHEREY-NAGEL GmbH & Co. KG, Düren, Germany) according to the manufacturer’s protocols. The quality and quantity of DNA were measured using a NanoDrop^®^ ND-1000 Spectrophotometer (Thermo Fisher Scientific, Waltham, MA, United States). The amount of total extracted DNA ranged from 5–143 ng/μl (mean 32 ± 7 (SE) ng/μl), and the DNA was stored at -20°C.

### Bacterial 16S rRNA Gene Amplification and 454 Sequencing

One hundred nanograms of purified genomic DNA from each sample were used as the template for PCR amplification. The V3-V6 hypervariable regions of the bacterial small-subunit (16S) rRNA genes were amplified with primers 338F (5′-ACT CCT ACG GGA GGC AGC AG-3′) (Weisburg et al., 1991) and 1046R (5′-CGA CAG CCA TGC ANC ACC T-3′) (Sogin et al., 2006) resulting in 726 bp of product size (*E. coli*, Access. No. U00096). In addition, the 5′ end of the forward primer was tagged with the Roche 454 pyrosequencing adapter “A” (5′-CGT ATC GCC TCC CTC GCG CCA TCA G-3′) as well as a unique 10 bp barcode sequence. For the reverse primer another adapter “B” (5′-CTA TGC GCC TTG CCA GCC CGC TCA G-3′) was used (Palmer et al., 2012). PCR amplification was performed in a total volume of 50 μL containing one-unit Phusion High-Fidelity DNA-Polymerase (Finnzymes Oy, Espoo, Finland), 10 μL buffer HF (5×), 1 μL of 10 mM of each dNTP, and 2.5 μL of primers (10 pmol μL^-1^). After an initial denaturation at 98°C for 30 s, there were 25 cycles of (1) 98°C for 10 s, (2) 67.8°C for 20 s, and (3) 72°C for 30 s, and a final extension at 72°C for 1 min. For each sample four PCR amplicons were generated, cut out from the agarose gel in the expected size range (800 bp), purified using the QIAquick gel extraction kit (Qiagen, Hilden, Germany), and pooled for the emPCR reaction. A standard concentration of 5 ng/μl was sequenced from both directions using 454 titanium chemistry (GS Junior, Roche, Basel, Switzerland) installed at the Red Cross Transfusion Service of Upper Austria (Linz, Austria). Four amplicons were sequenced in four regions each separated by 4-region gaskets, loading approximately 3,100,000 amplicon-coated beads per run and recovering a combined total of 800,000 sequence tags (Deng et al., 2017).

### Processing of Pyrosequencing Data and Statistics

Sequencing analysis was performed with the Quantitative Insights Into Microbial Ecology (QIIME, version 1.9.0) toolkit (Caporaso et al., 2010). Both forward (V4 region) and reverse (V6 region) sequences were processed and used as technical replicates. The clean reads were generated by *split_libraries.py* with default parameters (sequence length: 200–1000 bp, and average qual score: 25) to remove the potential adapters and primers and performing *identify_chimeric_seq.py* and *filter_fasta.py* to remove the chimeric sequences. After that, the clean reads were clustered to operational taxonomic units (OTUs) using an open reference strategy at the 97% similarity level (Stackebrandt et al., 2002; DeSantis et al., 2006). Subsequently, a representative sequence of each OTU was picked and assigned to a taxonomy based on the Greengenes Database (DeSantis et al., 2006).

Venn diagrams were constructed using jvenn (Bardou et al., 2014), and Circos plots were performed with R (Version 3.2.1). Variation in planktonic microbiota composition (beta-diversity) among samples was visualized via non-metric multidimensional scaling (NMDS) ordination based on Bray-Curtis dissimilarity matrices, while similarity of beta-diversity was tested via Analysis of Similarities (ANOSIM) using 999 permutations and Bray-Curtis distance. Both NMDS and ANOSIM were performed using the “vegan” package in R (Dixon, 2003). PCA and RDA were sequentially applied to relate OTUs abundance to the climatic and physico-chemical variables using CANOCO (version 4.5), Ter Braak and Smilauer, 2002: In a first step, since many of the environmental variables were autocorrelated, a Principal Component Analysis (PCA) was performed on the Pearson correlation matrix. Only the environmental variables that correlated most with PCA ordination axes 1-4 were kept for subsequent ordination analysis. These axes included monthly average water temperature (MAWT), YAWT, WAS, CiA, pH, Cl^-^, DOC, ${\mathrm{NO}}_{3}^{–}$ for the early growing season, and maximum depth, WAS, ${\mathrm{NO}}_{3}^{–}$, K^+^, Ca^2+^, TP, DOC, and DN for the late growing season. In a second step, from this subset of non-redundant variables redundancy analysis (RDA) using forward selection procedure was applied to select for the statistically significant environmental variables (*p* < 0.05). The potential dependence within consecutive samples taken repeatedly from the same lake was accounted for by including the sampling date as a covariate. Ordination biplots were constructed with CanoDraw. Alpha-diversity indices including richness (Chao1 and S_obs_, indicating estimated and observed OTUs) and diversity (Shannon-Weaver index and Simpson index) were calculated with packages “vegan” and “OTUtable” (Dixon, 2003). The richness indices Chao1 and S_obs_ were included into the model of MTE (Brown et al., 2004) with command *lm()*. MTE addresses the relationship between organismal metabolism and temperature using the formula (Alcaraz, 2016):

(1) $$l\,n\,\left(S_{c\,h\,a\,o\,1\,o\,r\,o\,b\,s}\right)=a-E_{a}\times \frac{1}{k\,T}$$

where *k* is Boltzmann’s constant (8.62 × 10^-5^ eV/K), and *T* is the absolute temperature in Kelvin (K). E_a_ is the activation energy, which equals the inverse of the slope calculated from the linear regression, and *a* is the intercept of the same linear regression.

To address the influence of water temperature more directly, functional genes (PFGs), such as metabolic genes, were predicted from the 16S rDNA derived OTUs using Phylogenetic Investigation of Communities by Reconstruction of States (PICRUSt, version 1.1.3) according to the Metagenome Prediction Tutorial (Langille et al., 2013) and related to the same units as applied for MTE. Mantel test, Pearson correlation, AIC value and significance analysis were performed with R. Specifically, Mantel test was used to estimate the correlation between the composition of bacterioplankton and environmental parameters with the package “vegan,” while Pearson correlation was used to investigate the environmental parameters and alpha-diversity indices correlation with the function *cor()*. AIC (Akaike information criterion) values were generated with the function *AIC()* to evaluate the goodness of fit to the linear regression. Significant differences of alpha-diversity indices among lakes were tested using one-way ANOVA (*p* < 0.05).

## Results

### Climate Change Effects and Environmental Parameters

The lakes had an average ice cover duration of 200 ± 18.7 (SD) days per year and maximum water temperature of 17.8°C (Supplementary Figures S1a,b). When comparing the two time periods 1998–1999 and 2009–2011 a trend of warming was observed. In particular, YAWT increased during the past decade by less than 1°C (Table 1). The measured increase in water temperature was related to increasing air temperatures as recorded from meteorological stations during the same observation period (see also Weckström et al., 2016). Compared with 1998–1999, ICDs in 2010–2011 were reduced by 11–37 days (Table 1). The shorter ICDs correlated significantly with the calendar day of spring mixing (R^2^ = 0.92), but not with the calendar day of autumn mixing (R^2^ = 0.007) (Supplementary Figures S1c,d). Consequently, the shorter ICD could be explained by an earlier ice break up in spring rather than by later ice formation in autumn.

The low concentrations of nutrients and Chl a in the study lakes indicated that all the five lakes were oligotrophic. The physico-chemical characteristics differed significantly among lakes because of differences in the geology of the various catchments (Kamenik et al., 2001), (Table 1). For example, OLA was slightly acidic (pH 6.3), while MOA was neutral (pH 7), and GIG, TWA, and WIR were slightly alkaline (pH 7.3–7.5). Furthermore, water chemistry such as ${\mathrm{NO}}_{3}^{–}$, ${\mathrm{SO}}_{4}^{2–}$, Cl^-^, Na^+^, K^+^, Mg^2+^, Ca^2+^, TP, DOC, and DRSi, differed significantly between lakes (*p* < 0.05, Table 1). During the study period, physico-chemical variables in each lake were found rather stable (Supplementary Table S1). Correlation analysis showed that environmental parameters were interrelated, e.g., in the early growing season, Cl^-^ showed high correlation with NH^4+^, Na^+^, K^+^, Mg^2+^, Ca^2+^, and TP (Supplementary Table S2). Not surprisingly, biological characteristics were found more variable. For example, cyanobacteria abundance ranged from 5.7 × 10^2^ to 2.3 × 10^5^ cells/ml. When compared with the early growing season, the bacterial abundance were found increased at the later stage of the growing season, i.e., in lake GIG, bacterial abundance was 1.78–1.94 × 10^6^ cells/ml in the early growing season but ranged from 2.51–2.90 × 10^6^ cells/ml in the late growing season. Altogether the results suggest that although study lakes differed in local influence, an on average minor, albeit significant increase in water temperature has become visible such as by reduced ice over duration.

### Composition of the Microbiota in the Alpine Lakes

After denoising, 161 943 sequences were obtained clustering into 13 050 OTUs as defined by the 97% identity threshold. The OTUs were assigned to 36 phyla. Among these, 14 phyla occurred in all 5 lakes and were considered core phyla. *Proteobacteria* were dominant, with relative abundances in the range of 48–75%. *Actinobacteria*, *Cyanobacteria*, *Bacteroidetes*, and *Firmicutes* were abundant with proportions of 3.1–26.7, 3.7–28.7, 6.9–19.7, and 0–8%, respectively (Supplementary Figures S2a,b). There were 11 pan phyla that occurred in one lake only and in lowest proportion (<0.01%, Supplementary Figure S2), i.e., the phyla WS5 in GIG; *Armatimonadetes*, *Fusobacteria* and SR1 in MOA; GN02 and OP11 in OLA; AD3 and AB3 in TWA; GN04, AC1 and an unknown phylum in WIR. The relative abundances of the dominant phyla varied by season, i.e., in the early growing season, the most abundant phyla included *Proteobacteria* (58.8%), *Cyanobacteria* (16.2%), *Actinobacteria* (10.6%), *Bacteroidetes* (6.4%), *Firmicutes* (5.3%), and *Acidobacteria* (1.2%), while in the late growing season, the dominant phyla were composed of *Proteobacteria* (61.8%), *Bacteroidetes* (17.2%), *Actinobacteria* (14.2%), and *Cyanobacteria* (5.7%) (Supplementary Figures S2c,d).

**FIGURE 2:**
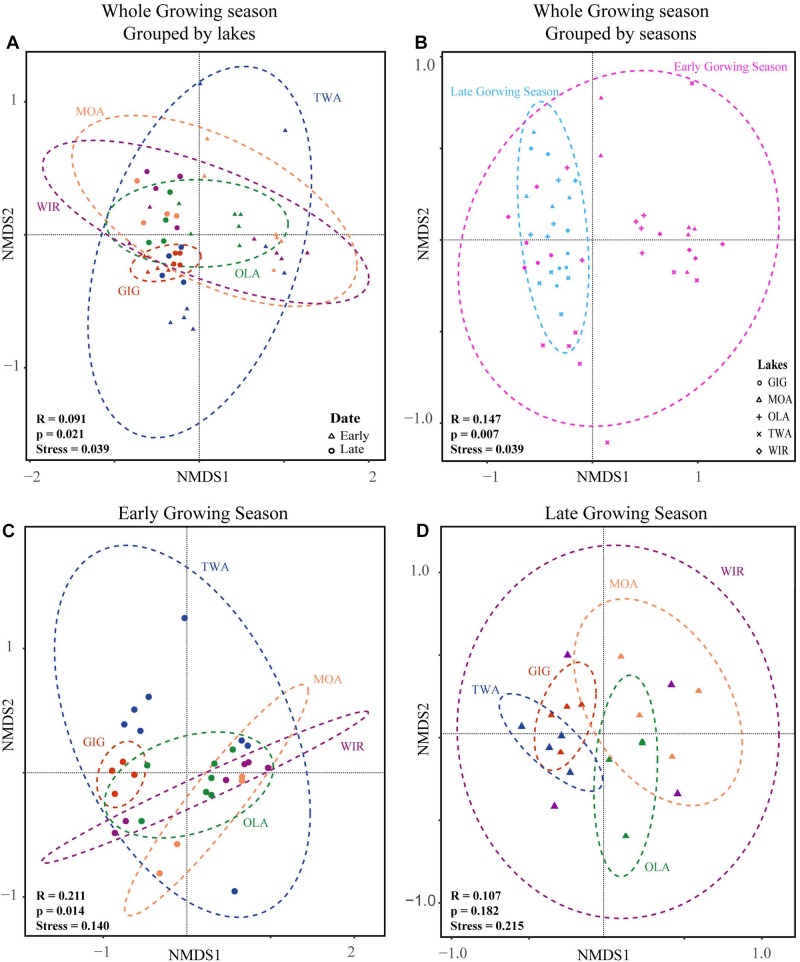
Non-metric multidimensional scaling (NMDS) plots of beta-diversity recorded from five alpine lakes using the Bray-Curtis matrix. **(A)** NMDS plots for individual lakes. **(B)** NMDS plots for samples from early and late growing season. **(C)** NMDS plots for individual lakes in the early growing season. **(D)** NMDS plots for individual lakes in the late growing season. Ellipses represent 95% confidence intervals (*p* < 0.05). ANOSIM (indicated by R and *p* values) revealed a significant dissimilarity between groups of individual lakes or growing seasons (*p* < 0.05).

**FIGURE 3:**
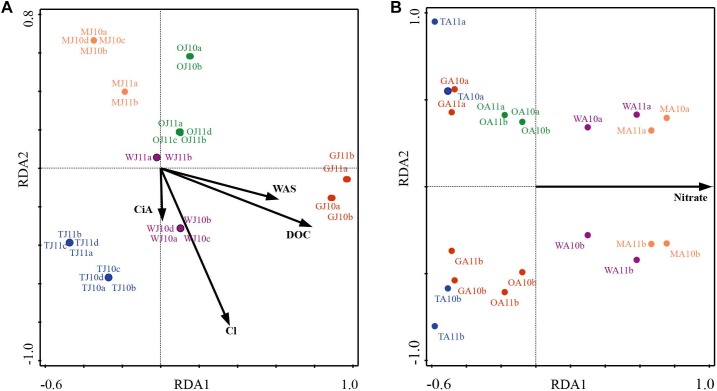
Redundancy analysis (RDA) of planktonic microbiota composition in five alpine lakes during the early growing season **(A)** and the late growing season **(B)**. The first characters indicates the lake name (G: GIG, M: MOA, O: OLA; T: TWA; W: WIR), and the second character indicates the sampling period (J: July, early stage of growing season, A: August, later stage of growing season).

NMDS analysis revealed a higher similarity of microbiota composition among lakes (Figure 2A), but higher variability between growing seasons (Figure 2B). Notably the microbiota showed higher variability in the early growing season (Figure 2C) than during the late growing season (Figure 2D). In the early growing season, planktonic microbiota structure was found significantly related to WAS, DOC, CiA, and Cl^-^, explaining 18.4% of the total inertia in OTU distribution as revealed by ordination analysis (Figure 3A). The influence of time dependence (included as a covariate) was found small and both the first axis and all four axes were statistically significant (Monte Carlo permutation tests, *n* = 499, *p* = 0.006, *p* = 0.002). Canonical axis 1 and 2 explained 6.5 and 5% of total OTU variation based on variables WAS and DOC vs. CiA and Cl^-^. In contrast, during the later growing season, only one variable (${\mathrm{NO}}_{3}^{–}$) explained 6.9% of the total OTU variation (Figure 3B). Using permutation analysis including time dependence as covariate the first axis was found marginally significant (*p* = 0.088). Correspondingly, Mantel tests revealed that for the early growing season, climatic parameters such as WAS and CiA, and nutrient concentrations were related to microbiota assembly. During later growing season, only nutrient concentrations, such as ${\mathrm{NO}}_{3}^{–}$ and TP were related to the microbial composition (Table 2). It is concluded that the microbiota composition during early growing season significantly depended on temperature and nutrients. In contrast during later season the influence of water temperature became less visible, while inorganic nutrients still played a significant role.

**Table 2:** Correlations between the planktonic microbiota composition in five alpine lakes and various environmental variables.

Early growing season			Late growing season		
Factor	r_(M)_	*p*	Factor	r_(M)_	*p*
**WAS**	**0.25**	**0.002**	Max depth	0.19	0.01
**CiA**	**0.27**	**0.002**	WAS	0.13	0.06
MAWT	0.08	0.061	**${\mathrm{NO}}_{3}^{–}$**	**0.25**	**0.01**
YAWT	0.15	0.006	Ca^2+^	–0.02	0.56
**Climate related variables**	**0.21**	**0.001**	K^+^	0.09	0.10
pH	0.14	0.004	**TP**	**0.22**	**0.02**
${\mathrm{NO}}_{3}^{–}$	0.19	0.003	DOC	0.13	0.04
Cl^-^	0.19	0.003	DN	0.14	0.03
DOC	0.18	0.002	**Nutrients**	**0.20**	**0.01**
**Nutrients**	**0.21**	**0.001**			


*Climate variables included WAS, CiA, MAWT, YAWT; Nutrients for the early growing season included ${\mathrm{NO}}_{3}^{–}$, Cl^-^, and DOC; Nutrients for the late growing season included ${\mathrm{NO}}_{3}^{–}$, Ca^*2*+^, K^+^, TP, DOC, and DN.*

### Richness and Diversity of Microbiota in Alpine Lakes

**FIGURE 4:**
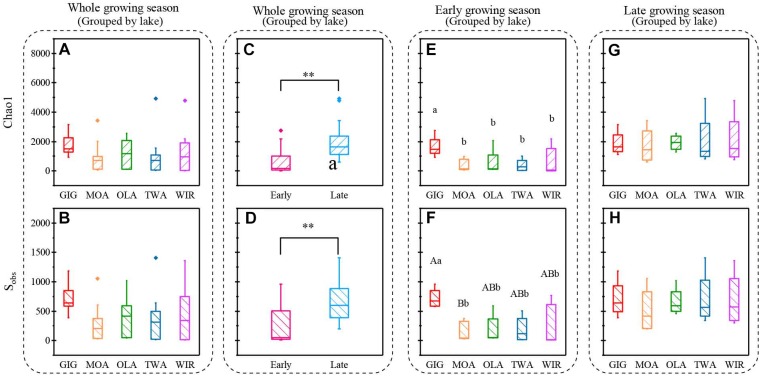
Indices of richness (a,c,e,g Chao1, b,d,f,h, S_obs_) recorded from planktonic microbiota for five alpine lakes **(A–D)** during the entire study period, **(E,F)** during early growing season, **(G,H)** during late growing season. Lowercase letters and ^∗^ indicate that subgroups differ (*p* < 0.05), while uppercase letters and ^∗∗^ indicate that subgroups differ at (*p* < 0.01).

Both richness and diversity differed spatially more among lakes in the early growing season, but less in the late growing season (Figure 4 and Supplementary Figure S3 and Supplementary Table S3). Seasonally α-diversity differed significantly between the two growing seasons, i.e., both richness and diversity indices were higher in the late growing season than in the early growing season (*p* < 0.01, Figure 4 and Supplementary Figure S3). Richness correlated with lake area, water temperature (WAS) and nutrients during the early growing season (Table 3). However, in the late growing season, correlation was found generally decreased and only ICD and CiA correlated with richness (Table 3). In contrast, diversity was significantly related to altitude, lake area, maximum depth, ICD, and CiA/CiS as well as nutrients. Using the same environmental variables as used for ordination analysis stepwise multiple regression included WAS, MAWT, pH, ${\mathrm{NO}}_{3}^{–}$, Cl^-^, YAWT for the prediction of richness indicator Chao1 (multiple R^2^ = 0.88, *p* < 0.001) and MAWT, pH, WAS, Cl^-^, ${\mathrm{NO}}_{3}^{–}$, and YAWT for S_obs_ (multiple R^2^ = 0.92, *p* < 0.001), (Supplementary Table S4). Vice versa for the prediction of diversity indices stepwise multiple regression included the variables CiA, MAWT, YAWT, WAS, pH, ${\mathrm{NO}}_{3}^{–}$, Cl^-^, DOC for Shannon (multiple R^2^ = 0.96, *p* < 0.001) and CiA, MAWT, YAWT, WAS, ${\mathrm{NO}}_{3}^{–}$, Cl^-^ for Simpson (multiple R^2^ = 0.86, *p* < 0.001 (Supplementary Table S4). It is concluded that besides lake morphometry, climatic variables related to water temperature and nutrients were influential to richness and diversity.

**Table 3:** Pearson correlation coefficients between environmental variables and α-diversity indices recorded from planktonic microbiota composition in five alpine lakes.

	Early growing stage				Late growing stage			
	Chao1	S_ob_	Shannon	Simpson	Chao1	S_ob_	Shannon	Simpson
Altitude	–0.08	–0.05	0.4*	0.43*	0	0.06	0.48*	0.41
Lake area	0.5**	0.56**	0.44*	0.24	–0.03	0.08	0.01	–0.15
Max depth^L^	–0.05	0.04	0.44*	0.48**	0.05	0.13	0.75***	0.62**

ICD	–0.38*	–0.35	0.24	0.43*	–0.51*	–0.5*	0.21	0.4
CiA^E^	0.39*	0.42*	0.4*	0.2	0.6**	0.72***	0.5*	0.09
CiS	–0.29	–0.25	0.36*	0.5**	–0.39	–0.36	0.34	0.44*
MAWT^E^	0.08	0.12	0	–0.13	–0.03	0.12	0.28	0.07
YAWT^E^	–0.01	–0.07	–0.3	–0.24	0.02	0.01	0.07	–0.02
WAS^EL^	0.55**	0.6***	0.47**	0.19	–0.02	0.13	0.27	0.03
WT	0.12	0.16	0.03	–0.11	–0.14	–0.01	0.35	0.19

Conductivity	0.17	0.26	0.13	0.11	0.06	0.11	0.42	0.28
pH^E^	–0.22	–0.16	–0.26	–0.25	–0.03	0.04	0.29	0.25
Alkalinity	0.2	0.29	0.12	0.09	0.08	0.13	0.39	0.24
${\mathrm{NO}}_{3}^{–EL}$	–0.14	–0.22	–0.55**	–0.53**	0.03	–0.08	–0.59**	–0.44*
${\mathrm{SO}}_{4}^{2–}$	0.05	0.16	0.31	0.34	0.04	0.1	0.63**	0.5*
Cl^-E^	–0.06	0.02	–0.33	–0.35	0.17	0.17	–0.09	–0.18
${\mathrm{NH}}_{4}^{+}$	0.23	0.39*	0.15	–0.13	0.32	0.33	–0.25	–0.3
Na^+^	–0.09	–0.04	–0.32	–0.26	–0.03	–0.09	–0.19	–0.1
K^+L^	–0.32	–0.44*	–0.48**	–0.38*	0.03	–0.06	–0.48*	–0.33
Mg^2+^	0.09	0.2	0.36	0.37*	0.09	0.16	0.67**	0.5*
Ca^2+L^	0.2	0.28	0.03	–0.01	0.09	0.14	0.28	0.14
TP^L^	–0.19	–0.07	–0.05	0.05	–0.34	–0.34	–0.02	0.13
DOC^EL^	0.51**	0.59***	0.61***	0.43*	0.24	0.36	0.23	–0.03
DN^L^	–0.46**	–0.52**	–0.47**	–0.09	–0.36	–0.39	–0.17	0.06
DRSi	–0.37*	–0.43*	–0.57***	–0.39*	–0.16	–0.3	–0.5*	–0.23


Statistical significance threshold (*p < 0.05, **p < 0.01, ***p < 0.005). ^*E*^parameters of the early growing season, ^*L*^parameters of the late growing season (as selected through PCA).

### Relationship Between Metabolic Activity and Temperature

**FIGURE 5:**
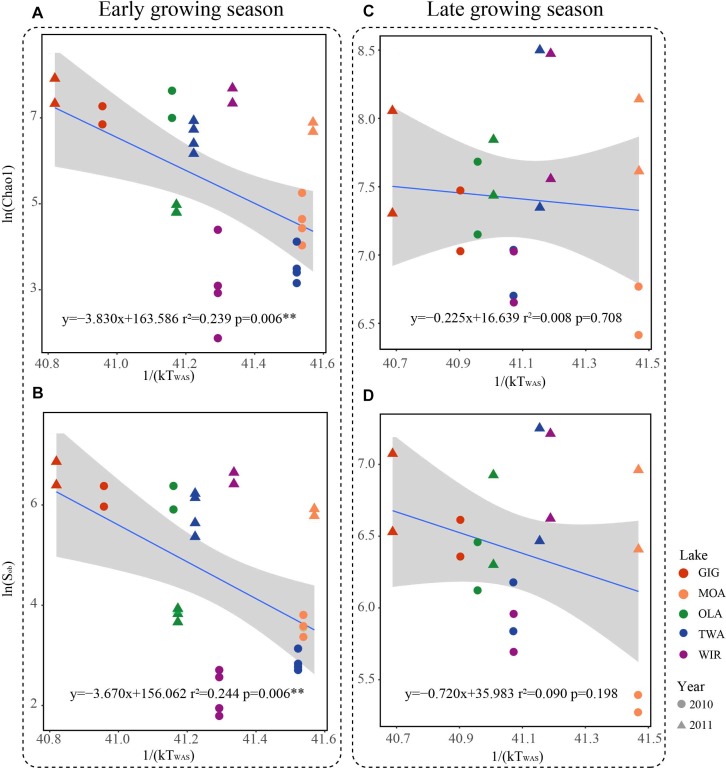
Relationships between taxonomic richness of microbiota in five alpine lakes and average water temperature after circulation in spring (WAS) in Kelvin (1/(KT_(WAS)_)). **(A)** Relationship between Chao1 and 1/(KT_(WAS)_) in the early growing season. **(B)** Relationship between S_obs_ and 1/(KT_(WAS)_) in the early growing season. **(C)** Relationship between Chao1 and 1/(KT_(WAS)_) in the late growing season. **(D)** Relationship between S_obs_ and 1/(KT_(WAS)_) in the late growing season.

Since multivariate ordination analysis revealed a significant influence of WAS and CiA on OTU abundance (Figure 3) and richness and diversity were found significantly related to water temperature (Table 2 and Supplementary Table S4) in the early growing season, we were interested to see to which extent MTE predicts higher richness and diversity with increasing water temperature. According to MTE only WAS (but not YAWT) was found significantly negatively related to richness in the early growing season (Figure 5 and Supplementary Figure S4). The activation energy as calculated from the inverse slope of the regression curve varied from -3.8 eV for Chao1 and -3.7 eV for S_obs_ exceeding the theoretical estimate for E_a_ of -0.65 eV (Brown et al., 2004). Notably in the later growing season the observed slopes were closer to the more frequently reported -0.65 prediction. In conclusion, while water temperature played an apparent role for metabolic activity during the period from ice break up to the first sampling date, this factor got less important later in the year.

### Functional Gene Prediction

**FIGURE 6:**
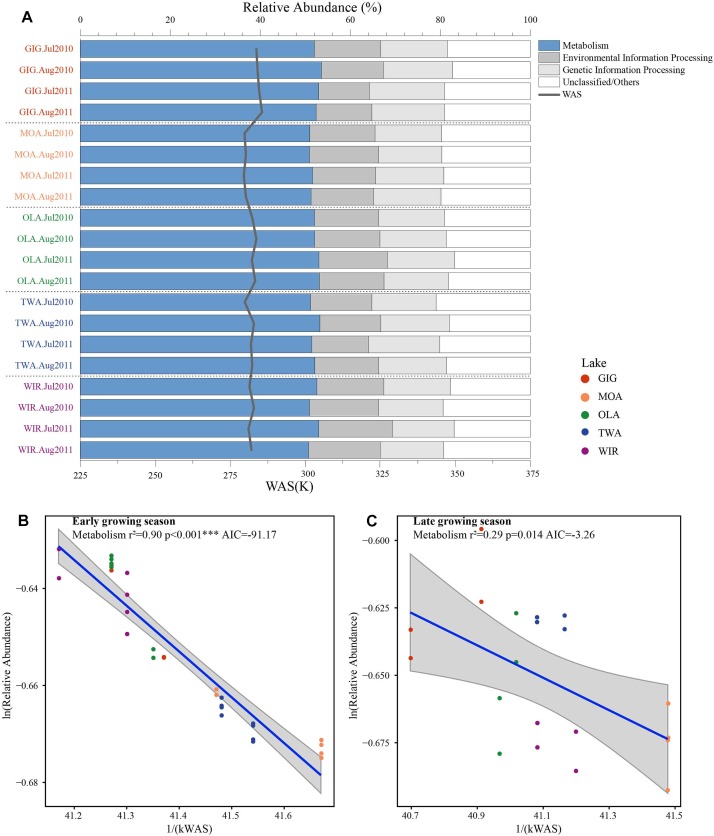
**(A)** Relative abundance of predicted functional genes in five alpine lakes and **(B)** relative abundance of metabolism related genes as a function of WAS in Kelvin (1/(KT_(WAS)_)) during early growing season and **(C)** later growing season.

To investigate the highlighted influence of water temperature more directly, functional genes (PFGs), such as metabolic genes, were predicted from the 16S rDNA derived OTUs using PICRUSt. Five groups of functional genes were predicted which included metabolism, environmental information processing, genetic information processing, others and unclassified. On average, metabolism related genes contributed the largest proportion that varied between and within habitats (Figure 6A). The natural logarithm of relative abundance of metabolism related genes [ln(PFGs-M)] was negatively related with 1/(kWAS) (Figure 6B) in the early growing season and also during the later growing season (Figure 6C). In summary, besides planktonic microbiota richness and diversity also metabolic genes increased proportional in response to water temperature supporting the more direct role of temperature variation in the study lakes.

## Discussion

### Microbiota in Remote Alpine Lakes

Microorganisms in alpine lakes are exposed to extreme environmental pressures such as high radiation, low temperature, short growing season, and low food availability (Sommaruga, 2001; Rose et al., 2009). Not surprisingly bacterial abundance, cyanobacterial abundance, and concentration of Chl a (Table 1) were lower than corresponding values in lowland lakes (Clasen et al., 2008) which is mostly because of lower trophic conditions and shorter vegetation times (Weckström et al., 2016). However, the abundances were in accordance with the microbial abundances reported for cold environments such as high altitude lakes in the Himalayan region (Sommaruga and Casamayor, 2009), the Qinghai-Tibet Plateau lakes (bacterial abundance ranged from 1.59 × 10^5^ to 3.37 × 10^5^ cells mL^-1^) and lakes in the Mount Everest region (Nepal, bacterial abundance ranged from 2.4 × 10^5^ to 1.15 × 10^6^ cells mL^-1^) (Liu et al., 2009; Sommaruga and Casamayor, 2009; Liu et al., 2011). The observed Chao1 and S_obs_ indices were similar to those reported for the bacterial community in lakes on Yunnan Plateau, while Shannon was lower (Chao1: 802∼2638, S_obs_: 579∼2005, average Shannon: 8.44; Zhang et al., 2015). The dominant phyla in the study lakes included *Proteobacteria*, *Actinobacteria*, *Cyanobacteria*, and *Bacteroidetes*, which accounted for 75–99.8% of the relative abundance, which is similar to the findings in Tibetan lakes and Nepal lakes (Sommaruga and Casamayor, 2009; Xiong et al., 2012). In contrast, abundances of other phyla, such as *Verrucomicrobia* and *Planctomycetes*, that have been reported frequently in lowland lakes (Newton et al., 2011; Gies et al., 2014; Zhang et al., 2015), were less abundant. In this study there was less difference spatially among lakes in composition or in diversity detected by NMDS or ANOVA when compared with seasonal variation (Figure 2, 4), indicating generally high similarity in structure and diversity of the alpine microbial communities at the spatial scale.

### Microbiota in Alpine Lakes and the Influence of Temperature

In this study, evidence showed that temperature and/or nutrients were drivers shaping the microbiota in the alpine lakes, which highlighted the potential sentinel role of alpine lakes for climate change related changes, i.e., after the ice break up during the early growing season (Figure 3 and Table 2). Both temperature and nutrients impact on the microbiota assembly directly and indirectly. For example, the nutrient level shapes microbiota both directly through bottom up regulation and indirectly through regulating the composition of the predators (Bouvy et al., 2011). Temperature after ice break up was shown to be a relevant factor determining microbial composition. Since about two dozen of environmental variables were found correlated in ordination analysis, the individual highlighted variables should be interpreted with caution although an indirect and direct increased temperature effect seems plausible. Direct effects would be related to higher metabolic activity and higher energy flux as outlined above. Indirect effects of increased temperature would include interactions among organisms in the lake food web, animal behavior, life histories, species interactions and ecosystem carbon cycling (Kraemer et al., 2017). Predator prey relations between zooplankton and bacteria (Kammerlander et al., 2016), bacteria and bacteria, such as *Cytophaga* vs. *Cyanobacteria*, and the viruses (Suttle, 2007; Bouvy et al., 2011; Martins et al., 2018) are also known as factors driving the assembly of microbiota in lakes. Besides direct effects it is known that the biomass and composition of plants are regulating the composition and diversity of planktonic bacteria in lowland lakes (Zeng et al., 2012) and may influence the seasonality of the pelagic microbiota in the alpine lakes as well. It seems possible that under increased terrestrial run off conditions during snow melt the influence from the catchment becomes more visible and contributes to the observed dependence of richness on temperature conditions. Terrestrial runoff can introduce organic matter and nutrients from soils in the catchment thereby structuring planktonic microbiota composition (Crump et al., 2012). In order to test the possibility that the richness or diversity may be influenced by run-off through rainfall or snow melting the precipitation recorded twice a day at two meteorological stations (Schmittenhöhe, 1956 m a SL, Obertauern, 1772 m) were compared for both seasonal periods. Average rainfall in 12 h was calculated from ice break up to the respective sampling date. In general, slightly higher average rainfall was recorded in the later season, i.e., in early vs. late growing season 2.1 ± 0.2 vs. 2.4 ± 0.2 mm rainfall in 12 h were recorded for Obertauern, and 2.5 ± 0.4 vs. 3.5 ± 0.2 mm rainfall in 12 h were recorded at Schmittenhöhe. In addition, the maxima of rainfall which were recorded varied between 22.1 vs. 24.1 mm during the early and late growing season for Obertauern, and 20.2 vs. 27.8 mm, respectively, for Schmittenhöhe. Thus, run-off influence on microbiota composition was considered stable when compared between both early and late growing periods.

### Richness of Alpine Planktonic Microbiota and the Influence of Water Temperature After Ice Break Up

Metabolic Theory of Ecology is a linear mechanistic approach addressing the correlation between metabolism of individuals, the population growth rate, the number of taxa and temperature. It underlines the role of temperature on the metabolic rate and taxon richness (Gillooly et al., 2001; Brown et al., 2004; Zhou et al., 2016). In general, for bacterial communities a few studies reported a significant dependence of richness vs. the inverse of annual average temperature. With their impressive example from soil Zhou et al. (2016) list numerous directly and indirect factors, mainly by annual average temperature, not at least by the influence of higher plant organisms. However, in this study, we found that the annual average temperature (YAWT) did not contribute significantly as explanatory variable in alpine lakes (Supplementary Figure S4) which would rather argue against a potential influence related to specific habitat characteristics (e.g., so called under cooled lakes, Thompson et al., 2005). On the contrary to YAWT, WAS showed significant correlation with richness (Figure 5), indicating the potential of water temperature to regulate the microbial metabolic rate in the early growing season after ice break up. Additionally, the significant correlation between the WAS and proportion of metabolic genes (Figure 6) implies that bacteria contributing to metabolic activity increased along with temperature. Notably, activation energy (E_a_), as calculated from the slope and reflecting the metabolic rate, was -3.83 for Chao1 and -3.67 for S_obs_ exceeding the more frequent estimate five times (Brown et al., 2004) (Figure 5). In other words, the influence of temperature was more visible when compared with microbiota analyzed from lower altitudes. Compared with lowland lakes the microbiota in alpine lakes suffers from a shorter growing season that typically lasts from June to October (Parker et al., 2008). It might be speculated that during the early period after ice break up energy limitation of metabolic growth is the dominant factor while availability of nutrients or light availability is of minor importance. It is generally known that colder (arctic and alpine) habitats are warming faster than warmer places and warming phases in spring become more intense. The water phase in colder places might show steeper increases and perhaps higher amplitudes for a given time window when compared with habitats less affected by climate change. Steeper increases or decreases in temperature for a certain time period might explain the rather steep slopes observed in the early growing phase after ice break up. Vice versa after a certain temperature threshold is exceeded the limiting role of temperature gets less important as the seasonal vegetation period proceeds and nutrients and perhaps biotic factors become more relevant.

## Conclusion

Within the 10 years period climate change was visible through reduced ice cover duration and increased average water temperature. While WAS and CiA, as well as nutrients and DOC, had a significant influence on bacterial community composition during the early growing season, only nutrients (such as nitrate) were found influential later in the growing season. In other words, the limiting role of temperature became overruled by limiting nutrients later in the growing season. MTE could explain the dependence of taxonomic richness on bacterial metabolic activity in mathematical terms. Interestingly the activation energy exceeded the MTE predicted estimate by far emphasizing the dominant role of temperature during early growing season. In contrast later in the growing season a less significant MTE dependency could be observed. The dominant influence of temperature after ice break up could be explained by overall climate change effects, such as a more intense warming in spring and an overall higher amplitude of temperature variation.

## Data Availability

The datasets generated for this study can be found in NCBI Raw Sequence Read Archive (SRA), SRP181537.

## Author Contributions

RK and SB collected the samples and the metadata as well as extracted DNA and performed the sequencing. YJ, LD, RK, and HH analyzed and interpreted the data, and drafted the manuscript. YJ performed most of the bioinformatic and statistical analyses. JR assisted in bioinformatic analysis. YJ and HH constructed the figures and tables. TM uploaded the sequences. All authors approved the final version of the manuscript for submission and agreed to be accountable for the work.

## Conflict of Interest Statement

The authors declare that the research was conducted in the absence of any commercial or financial relationships that could be construed as a potential conflict of interest.
