# Supplementary material for: Temperature Response of Planktonic Microbiota in Remote Alpine Lakes
Source: Front Microbiol. 2019 Jul 31;10:1714. doi: 10.3389/fmicb.2019.01714 (PMC6685043; doi:10.3389/fmicb.2019.01714)
Supplement: Supplementary file 1 [file Data_Sheet_1.docx]

***Supplementary materials***

**Temperature response of planktonic microbiota in remote alpine lakes**

Yiming Jiang ^1, 2^, Haiying Huang ^1, 2^, Tianli Ma ^1, 2^, Jinlong Ru ^1,2^, Stephan Blank^3^, Rainer Kurmayer ^3, *^, Li Deng ^1, 2, *^

1. Institute of Virology, Helmholtz Zentrum München, German Research Center for Environmental Health, Neuherberg, Germany

2. Institute of Virology, Technical University of Munich, München, Germany

3. Research Department for Limnology, Mondsee, University of Innsbruck, Mondseestrasse 9, 5310 Mondsee, Austria

*Corresponding Author.

Rainer Kurmayer (e-mail: [rainer.kurmayer@uibk.ac.at](mailto:rainer.kurmayer@uibk.ac.at))

Li Deng (e-mail: li.deng@helmholtz-muenchen.de)

Telephone: 0043-512-507-50242

**Abbreviations list**

| **Terms** | **Abbreviations** |
| --- | --- |
| 1. **Lakes** | |
| Unterer Giglachsee | GIG |
| Moaralmsee | MOA |
| Oberer Landschitzsee | OLA |
| Twenger Almsee | TWA |
| Wirpitschsee | WIR |
| 1. **Environmental parameters** | |
| Calendar day of circulation in autumn () | CiA |
| Calendar day of circulation in spring | CiS |
| Duration of ice cover | ICD |
| Annual average water temperature | YAWT |
| Monthly average water temperature | MAWT |
| Average water temperature between calendar day of circulation in spring until the sampling date | WAS |
| Conductivity | Cond. |
| Chlorophyll a | chl-a |
| Sulphate | SO_4_^2-^ |
| Chloride | Cl^-^ |
| Ammonium | NH_4_^+^ |
| Sodium | Na^+^ |
| Potassium | K^+^ |
| Magnesium | Mg^2+^ |
| Calcium | Ca^2+^ |
| Total phosphorus | TP |
| Dissolved organic carbon | DOC |
| Nitrate | NO_3_^-^ |
| Dissolved nitrogen | DN |
| Dissolved reactive silica | DRSi |
| 1. **Bioinformatic and statistic terms** | |
| Operational taxonomic units | OTUs |
| Non-metric multidimensional scaling | NMDS |
| Analysis of Similarities | ANOSIM |
| Principal component analysis | PCA |
| On-redundant variables redundancy analysis | RDA |
| S_obs_ | Observed species |
| Functional genes | PFGs |
| 1. **Others** | |
| Glass fibre filters | GF/C |
| Nitrocellulose membranes | NC |
| Above sea level | a.S.L. |


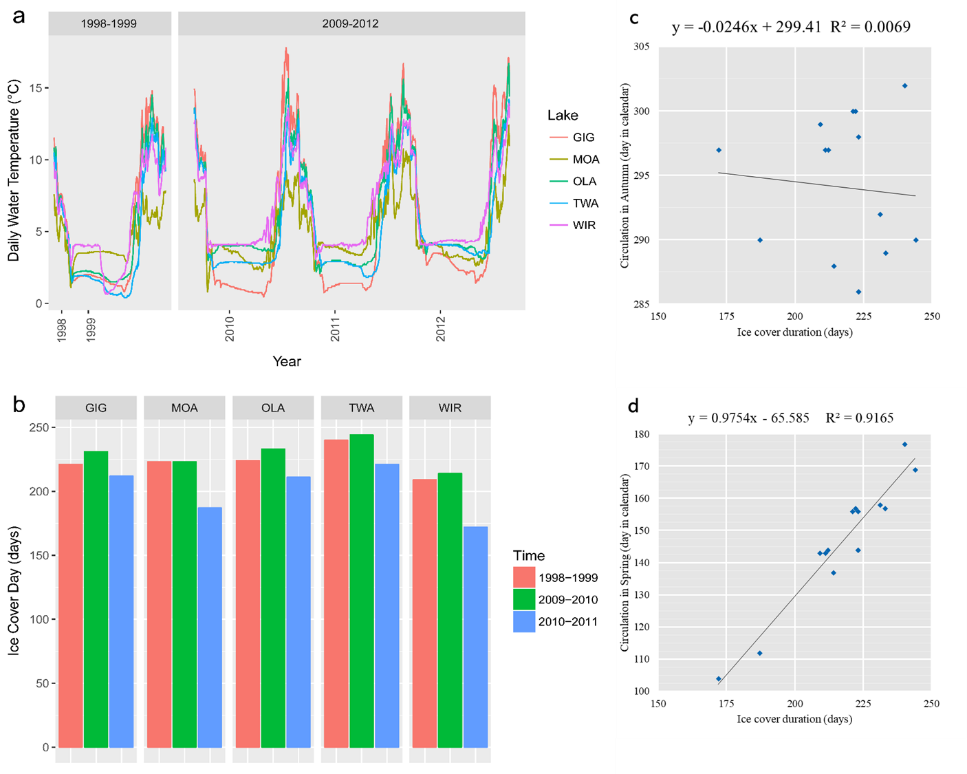


**Supplementary Fig. 1** (a) Daily water temperature in five alpine lakes located in the “Niedere Tauern” region of the Austrian Alps (GIG, MOA, OLA, TWA and WIR). Water temperatures were monitored in two periods. The first period started from September 5th, 1998 and ended on September 26th, 1999; the second period was from September 1st, 2009 to August 27th, 2012. (b) Ice cover duration in days of the five lakes during the periods of 1998-1999, 2009-2010, and 2010-2011. (c,d) Scatter plots between ice cover duration in days and (c) day of circulation in Autumn (day in calendar) and (d) day of circulation in Spring (day in calendar) and calculated linear regression curves.


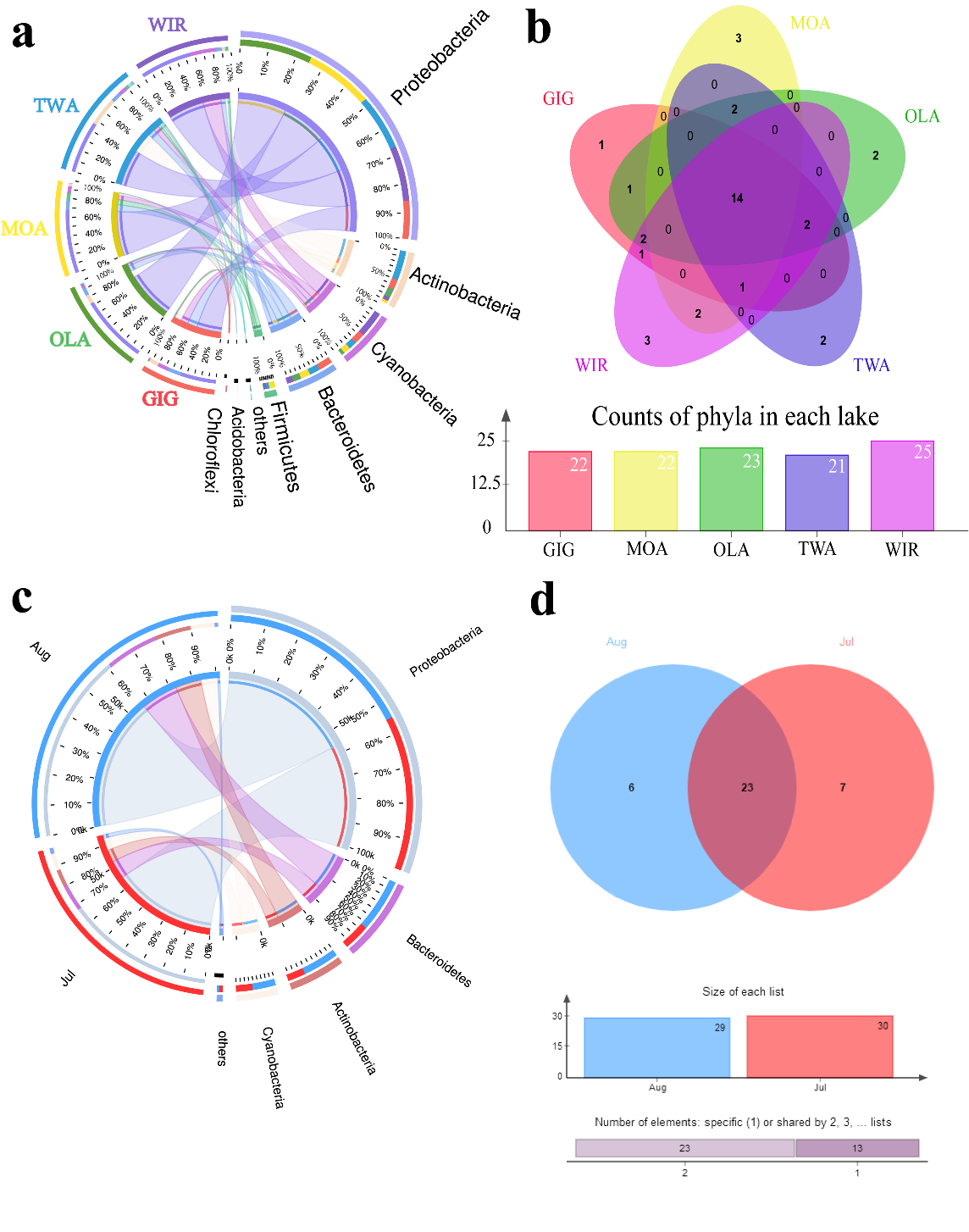


**Supplementary Fig. 2.** Composition of bacterioplankton community in five alpine lakes at phylum level. (a) Circos plot showing bacterioplankton at phylum level grouped according to study lake. (b) Venn (upper panel) shows core and pan bacterioplankton in lakes; Bar chart (lower panel) shows counts of phyla in each lake. (c) Circos plot showing bacterioplankton at phylum level grouped according to early and late growing season. (d) Venn (upper panel) shows core and pan bacterioplankton in early and late growing seasons; Bar chart (lower panel) shows counts of phyla in each growing season.


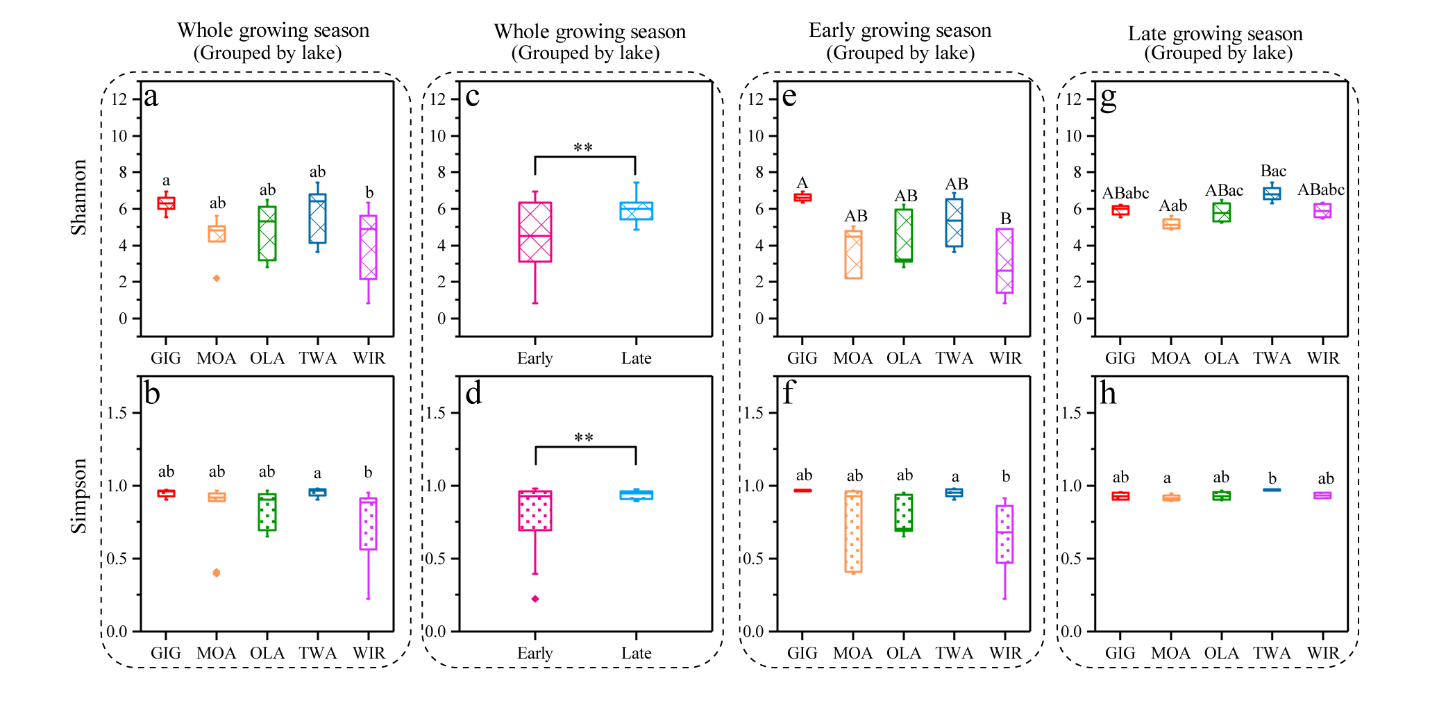


**Supplementary Fig. 3**. Indices of diversity (a,c,e,g Shannon, b,d,f,h, Simpson) recorded from planktonic microbiota in five alpine lakes (a,b,c,d) during the entire study period, (c,f) during early growing season, (g,h) during late growing season. Lowercase letters and * indicate that subgroups differ (p<0.05), while uppercase letters and ** indicate that subgroups differ at (p<0.01).


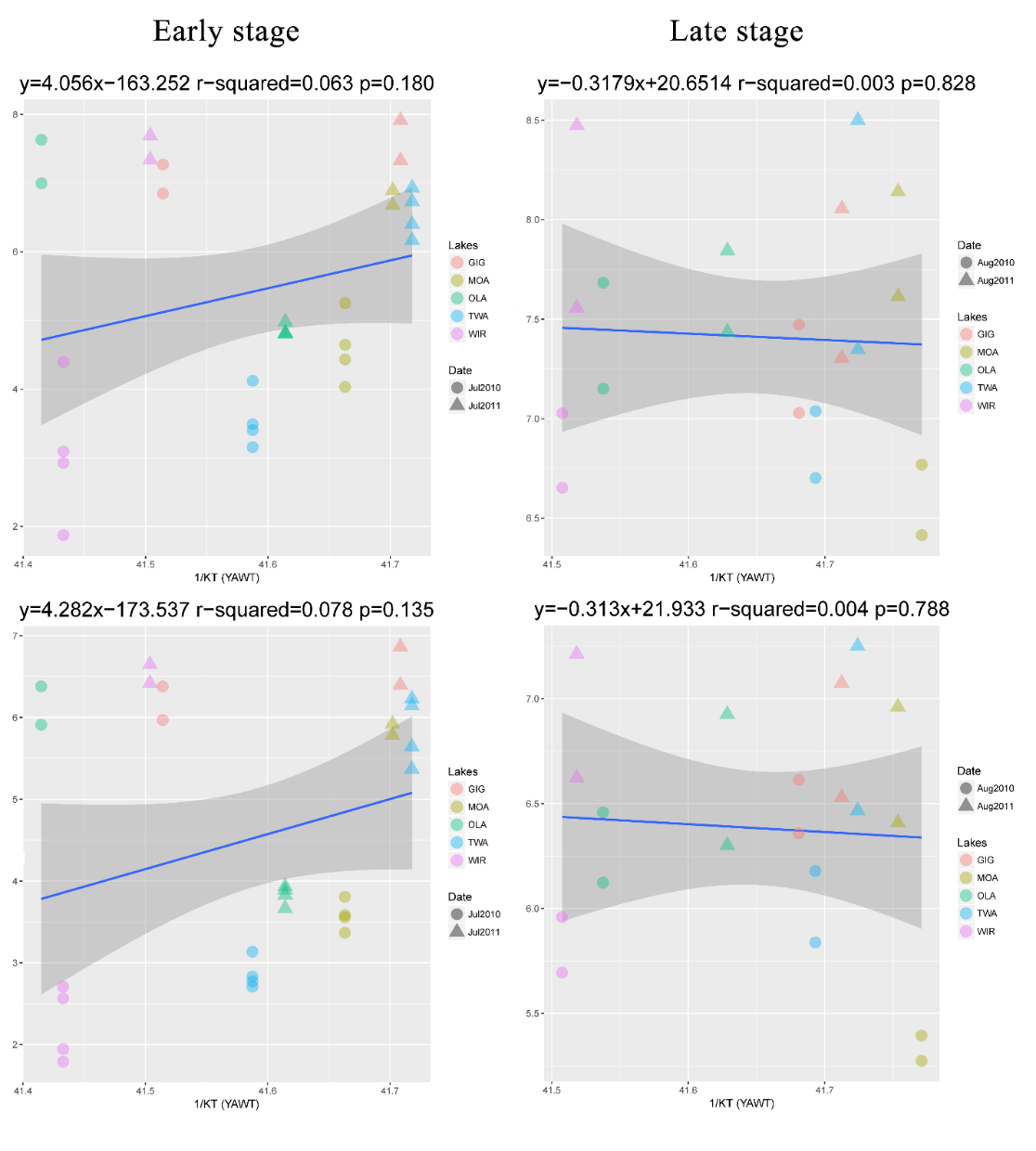


**Supplementary Fig. 4.** Relationships between richness for planktonic microbiota and yearly average water temperature in Kelvin (1/KT_(YAWT)_) observed in five alpine lakes. Upper panel: Scatter plots between Chao and 1/(kT_YAWT_) in the early and late growing season, Lower panel: Scatter plots between S_obs_ and 1/(kT_YAWT_) in the early and late growing season.

**Supplementary Table 1.** Environmental variables recorded in five alpine lakes of the “Niedere Tauern” region in the Austrian Alps

| Sample | Cond. | pH | Alkalinity | Nitrate | Sulfate | Cl | Ammonium | Na | K | Mg | Ca | TP | DOC | DN | DRSi | Chla | Bacteria | Cyanobacteria |
| --- | --- | --- | --- | --- | --- | --- | --- | --- | --- | --- | --- | --- | --- | --- | --- | --- | --- | --- |
|  | (μS/cm) |  | (µeq/L) | (µM) | (µM) | (µM) | (µM) | (µM) | (µM) | (µM) | (µM) | (µM) | (µg/L) | (µg/L) | (µM) | (µg/L) | counts/ml | counts/ml |
| GIG_Jul2010 | 73.8 | 6.92 | 598 | 0.29 | 61.2 | 4.75 | 0.33 | 19.09 | 3.82 | 90.29 | 270.55 | 0.18 | 873 | 88 | 10.47 | NA | 1.78E+06 | 3.22E+04 |
| GIG_Aug2010 | 74.9 | 7.92 | 607 | 0.1 | 60.99 | 5.12 | 0.11 | 18.96 | 3.54 | 90.58 | 266.95 | 0.15 | 759 | 50 | 9.84 | 2.4 | 2.51E+06 | 5.54E+04 |
| GIG_Jul2011 | 74.1 | 7.59 | 625 | 0.27 | 60.63 | 4.53 | 0.28 | 18.48 | 3.44 | 94.04 | 273.05 | 0.1 | 931 | 73 | 8.75 | 1.8 | 1.94E+06 | 1.02E+05 |
| GIG_Aug2011 | 73.8 | 7.57 | 600 | 0.24 | 59.61 | 4.75 | 0.22 | 19.52 | 4.05 | 94.08 | 276.28 | 0.12 | 869 | 83 | 7.79 | 1.8 | 2.90E+06 | 2.32E+05 |
| MOA_Jul2010 | 26.9 | 7.35 | 174 | 2.95 | 26.96 | 2.32 | 0.11 | 20.26 | 6.97 | 11.29 | 104.48 | 0.1 | 445 | 205 | 19.72 | NA | 5.79E+05 | 1.03E+03 |
| MOA_Aug2010 | 31.8 | 6.13 | 198 | 3.13 | 31.88 | 4.35 | 0.06 | 22.43 | 7.62 | 12.46 | 116.8 | 0.13 | 424 | 198 | 19.59 | 1.3 | 7.07E+05 | 1.23E+03 |
| MOA_Jul2011 | 28 | 7.37 | 181 | 3.35 | 28.44 | 4.08 | 0.22 | 20.7 | 7.05 | 11.63 | 115.15 | 0.08 | 378 | 212 | 17.77 | 0.02 | 5.79E+05 | 5.70E+02 |
| MOA_Aug2011 | 28.6 | 6.93 | 194 | 3.1 | 28.89 | 4.43 | 0.5 | 22.13 | 7.74 | 12 | 114.23 | 0.1 | 532 | 221 | 19.52 | 0.04 | 1.09E+06 | 1.02E+03 |
| OLA_Jul2010 | 14.1 | 5.73 | 66 | 1.08 | 20.65 | 2.27 | 0.11 | 12.13 | 7 | 5.75 | 46.45 | 0.06 | 651 | 109 | 11.22 | NA | 4.15E+05 | 6.66E+02 |
| OLA_Aug2010 | 15.1 | 6 | 69 | 1.06 | 21.77 | 2.43 | 0.06 | 12.26 | 7.08 | 5.67 | 46.15 | 0.09 | 586 | 93 | 11.28 | 0.5 | 9.48E+05 | 5.19E+02 |
| OLA_Jul2011 | 13.6 | 6.46 | 75 | 1.03 | 22.19 | 3.81 | 0.28 | 14.87 | 7.49 | 6.04 | 53.85 | 0.07 | 603 | 128 | 10.5 | 0.1 | 5.74E+05 | 2.82E+02 |
| OLA_Aug2011 | 13.8 | 6.57 | 72 | 1 | 22.02 | 3.15 | 0.22 | 13.26 | 7.33 | 5.88 | 51.05 | 0.08 | 684 | 107 | 10.35 | 0.6 | 1.05E+06 | 4.18E+02 |
| TWA_Jul2010 | 73.9 | 7.24 | 560 | 0.05 | 80 | 4.67 | 0.17 | 19.43 | 4.82 | 125.88 | 228.55 | 0.13 | 576 | 383 | 13.7 | NA | 1.08E+06 | 9.40E+03 |
| TWA_Aug2010 | 73.2 | 7.4 | 553 | 0 | 76.8 | 3.81 | 0.11 | 18.78 | 4.49 | 120.29 | 220.53 | 0.13 | 540 | 390 | 12.96 | 1.1 | 2.23E+06 | 2.33E+04 |
| TWA_Jul2011 | 71.4 | 7.46 | 552 | 0.06 | 75.32 | 3.6 | 0.28 | 18.7 | 4.64 | 123.96 | 225.23 | 0.08 | 619 | 49 | 9.77 | 0.2 | 2.13E+06 | 6.29E+03 |
| TWA_Aug2011 | 71.1 | 7.33 | 546 | 0 | 73.15 | 3.57 | 0.17 | 19.3 | 4.92 | 123.54 | 224.78 | 0.09 | 626 | 52 | 9.3 | 1.3 | 2.12E+06 | 7.98E+03 |
| WIR_Jul2010 | 83 | 8.07 | 693 | 2.58 | 52.33 | 5.31 | 0.22 | 23.87 | 5.49 | 66.88 | 332.43 | 0.13 | 518 | 178 | 18.56 | NA | NA | NA |
| WIR_Aug2010 | 84.8 | 7.87 | 724 | 2 | 52.94 | 4.96 | 0.33 | 28.17 | 4.28 | 70.67 | 342.38 | 0.06 | 141 | 435 | 19.12 | 0.3 | 8.65E+05 | 1.41E+03 |
| WIR_Jul2011 | 88.2 | 7.12 | 760 | 2.61 | 52.64 | 4.19 | 0.22 | 24.87 | 5.82 | 71.75 | 361.05 | 0.08 | 510 | 173 | 16.84 | 0.4 | 5.97E+05 | 1.44E+03 |
| WIR_Aug2011 | 85 | 6.77 | 722 | 2.89 | 52.78 | 6.75 | 0.28 | 24.65 | 5.33 | 70.5 | 354.93 | 0.06 | 459 | 192 | 16.86 | 0.3 | 1.28E+06 | 5.94E+03 |

**Supplementary Table 2.**  Correlations between environmental variables recorded in five alpine lakes during early and late growing season.

1. **Early growing season**
2. **Late growing season**

“Supplementary Table 2.xlsx”

“***” means p < 0.005, “**” means p < 0.01, “*” means p < 0.05, “+” means p < 0.1, and “.” means p < 0.5

**Supplementary Table 3.** Alpha-diversity recorded from planktonic microbiota in five alpine lakes.

|  | Chao1 | S_obs_ | Shannon | Simpson |
| --- | --- | --- | --- | --- |
| GIG.Jul2010 | 1188.88 | 661 | 6.75 | 0.97 |
| GIG.Aug2010 | 1443.50 | 490 | 6.17 | 0.95 |
| GIG.Jul2011 | 2131.79 | 779 | 6.52 | 0.96 |
| GIG.Aug2011 | 2319.52 | 933 | 5.71 | 0.91 |
| MOA.Jul2010 | 108.84 | 36.25 | 4.69 | 0.95 |
| MOA.Aug2010 | 740.37 | 207.5 | 5.06 | 0.92 |
| MOA.Jul2011 | 889.89 | 349 | 2.20 | 0.40 |
| MOA.Aug2011 | 2728.78 | 831 | 5.31 | 0.91 |
| OLA.Jul2010 | 1577.19 | 479.5 | 6.10 | 0.94 |
| OLA.Aug2010 | 1723.12 | 547 | 5.94 | 0.93 |
| OLA.Jul2011 | 128.23 | 46.25 | 3.10 | 0.69 |
| OLA.Aug2011 | 2123.58 | 781.5 | 5.68 | 0.93 |
| TWA.Jul2010 | 36.92 | 17.75 | 3.95 | 0.92 |
| TWA.Aug2010 | 976.26 | 412.5 | 6.56 | 0.97 |
| TWA.Jul2011 | 734.77 | 367.25 | 6.58 | 0.97 |
| TWA.Aug2011 | 3233.03 | 1026.5 | 7.11 | 0.97 |
| WIR.Jul2010 | 32.03 | 10.25 | 1.87 | 0.51 |
| WIR.Aug2010 | 950.24 | 342 | 5.55 | 0.93 |
| WIR.Jul2011 | 1861.49 | 692.5 | 4.90 | 0.89 |
| WIR.Aug2011 | 3351.07 | 1055 | 6.26 | 0.93 |

**Supplementary Table 4.** Richness indices (Chao1, S_obs_) for planktonic microbiota in five alpine lakes predicted by environmental parameters during the early growing season as calculated from forward stepwise regression analysis.

1. **Chao1**

| **Coefficients** | **Estimate** | **p** |
| --- | --- | --- |
| WAS | 962.574 | 1.11E-11 *** |
| MAWT | -532.909 | 5.25E-10 *** |
| pH | -1331.934 | 2.61E-07 *** |
| NO_3_^-^ | 5.225 | 4.77E-05 *** |
| Cl^-^ | 9407.872 | 0.00151 ** |
| YAWT | 350.973 | 0.00555 ** |
| (Intercept) | 4586.224 | 0.00199 ** |
| Multiple R-squared | 0.8789 | |
| p-value | 1.898^-9^ | |

1. **S_obs_**

| **Coefficients** | **Estimate** | **p** |
| --- | --- | --- |
| MAWT | -228.3370 | 6.31E-09 *** |
| pH | -543.3595 | 2.70E-07 *** |
| WAS | 489.0243 | 1.48E-06 *** |
| Cl^-^ | 4589.5959 | 9.14E-05 *** |
| NO_3_^-^ | 1.1393 | 0.0366 * |
| YAWT | 68.3622 | 0.0895 |
| CiA | -15.9632 | 0.616 |
| (Intercept) | 6866.3450 | 0.0203 * |
| Multiple R-squared | 0.924 | |
| p-value | 4.636^-10^ | |

1. **Shannon**

| **Coefficients** | **Estimate** | **p** |
| --- | --- | --- |
| CiA | -0.1557 | 1.26E-03 ** |
| MAWT | -1.0201 | 7.21E-08 *** |
| YAWT | 0.5298 | 1.44E-02 * |
| WAS | 2.511 | 1.65E-06 *** |
| pH | -1.0788 | 9.65E-03 ** |
| NO_3_^-^ | -0.0152 | 9.87E-06 *** |
| Cl^-^ | -17.0848 | 2.29E-03 ** |
| DOC | -0.0083 | 1.37E-02 * |
| (Intercept) | 54.7372 | 8.89E-04 *** |
| Multiple R-squared | 0.9463 | |
| p-value | 1.292E-11 | |

**(d) Simpson**

| **Coefficients** | **Estimate** | **p** |
| --- | --- | --- |
| CiA | -0.0108 | 1.26E-03 ** |
| MAWT | -0.068 | 7.21E-08 *** |
| YAWT | 0.1038 | 1.44E-02 * |
| WAS | 0.1005 | 1.65E-06 *** |
| NO_3_^-^ | -0.002 | 9.65E-03 ** |
| Cl^-^ | -3.5016 | 9.87E-06 *** |
| (Intercept) | 4.0001 | 8.89E-04 *** |
| Multiple R-squared | 0.7418 | |
| p-value | 8.476e-06 | |
